# Comparing Entomology-Themed Outreach Events: Annual Festivals and Open Houses in the United States

**DOI:** 10.3390/insects15050337

**Published:** 2024-05-07

**Authors:** Stephanie Blevins Wycoff, Daniel L. Frank, Michael J. Weaver

**Affiliations:** Department of Entomology, College of Agriculture and Life Sciences, Virginia Tech, Blacksburg, VA 24061, USA; dlfrank@vt.edu (D.L.F.); mweaver@vt.edu (M.J.W.)

**Keywords:** entomology outreach events, insect festivals, arthropods

## Abstract

**Simple Summary:**

Entomology festivals and open houses seek to educate the public about insects and other arthropods and have been growing in popularity over the past several decades. These events aim to increase understanding and appreciation for these often misunderstood animals. A study was initiated in 2016 to identify science institutions across the United States hosting these public outreach events. Literature reviews and online searches revealed several institutions hosting entomology-themed outreach events, which were subsequently contacted to participate in a survey regarding their events. The survey received a response rate of 93%. Results from the survey offered valuable insights into these events, including records of attendance, event structure, funding sources, popular exhibits, and the impacts on attendees, hosting institutions, and local communities. Many events experienced significant disruptions due to the COVID-19 pandemic, prompting some to transition to online experiences. Despite these challenges, most of these events have remained in place since the survey, highlighting the continued commitment to entomological outreach among these institutions.

**Abstract:**

Over the past several decades, there has been a growing prevalence of entomology-themed outreach events, which seek to educate the public about insects and other arthropods, fostering a greater appreciation and understanding of these often misunderstood organisms. In 2016, a comparative analysis was initiated to identify science institutions across the United States engaged in providing annual entomology-themed outreach events to the public. Utilizing literature reviews and online searches, several science institutions were identified and subsequently contacted to partake in a survey regarding their events. The survey received a response rate of 93%. Results from the survey offered valuable insights into these entomology-themed outreach events, including records of attendance, event structures, funding sources, popular exhibits, and the impacts on attendees, hosting institutions, and local communities. While the majority of these events have remained in place since the survey, many have experienced significant disruptions due to the COVID-19 pandemic, prompting some to adapt to innovative online formats and virtual experiences. Despite these challenges, the commitment to entomological outreach continues today, highlighting the resilience and adaptability of the entomology community.

## 1. Introduction

Throughout the United States and various other countries, there exist numerous entomology-themed outreach events dedicated to the appreciation of insects and other arthropods. Several of these events have been ongoing since the 1980s, with their popularity steadily growing [1,2]. Despite this trend, studies show that the general public often harbors negative feelings toward these animals [3]. Kellert [4] reported significant antipathy and disgust toward insects and spiders among Connecticut residents, while Davey [5] found consistent fears of cockroaches, spiders, and wasps among residents of the United Kingdom. Other studies have indicated that the external morphology of arthropods (vastly different from that of humans) shapes people’s perceptions of these animals [6,7]. Consequently, a common objective of entomology-themed outreach events has been to educate the public about the significance of insects and other arthropods and their positive impact on our lives [1,8]. In addition to education, other objectives often include entertainment, celebration, and conservation of specific species [1,9,10].

Previous research studies have compiled data on large entomology-themed outreach events. One study examined entomology festivals at both national and international levels, documenting details such as attendance records, the inclusion of arthropods other than insects, and the objectives of these events [1]. Similarly, another study focused on entomology festivals within North America and provided analogous information [9]. Furthermore, a study conducted in Japan cataloged numerous festivals dedicated to a single insect, most commonly fireflies and rhinoceros beetles [10]. Although the literature is limited, these studies indicate the existence of numerous well-established entomology-themed outreach events worldwide that appear to have a strong presence within their communities.

Since insects and other arthropods are often considered “aversive taxa”, entomologists face challenges in communicating with the public [2,3]. To counteract negative attitudes, entomology-themed outreach events often incorporate live arthropods and pinned insect collections, which are attractive to attendees and provide an opportunity for close viewing of these animals [11,12,13]. This practice, combined with educational exhibits and interactions with entomologists, is thought to raise awareness about the importance of insects and other arthropods [14,15].

Although literature reviews and web searches reveal the existence of many entomology-themed outreach events [8,11,12], few published studies have been conducted to assess their impacts [2,13,16]. Here, we discuss the results of a survey used to assess the purpose and organization of major entomology-themed outreach events in the United States and the impacts they have on their audiences, institutions, and surrounding communities.

## 2. Materials and Methods

In 2016, an online University Entomology Outreach Events (UEOE) survey was developed in Qualtrics (Provo, UT, USA) to attain information about entomological-themed events at science institutions throughout the United States [PDF File: S1]. The survey consisted of 19 questions categorized into five sections: event information, attendee demographics and information, attendee attitudes toward arthropods and insects, community/institutional impacts of the event, and interest in collaboration. The majority of questions were closed-ended, with two sections of the survey utilizing Likert-type scale statements. The Virginia Tech Institutional Review Board granted an exemption for the data collection (VT IRB no. 16-587).

A list of major entomology-themed outreach events in the United States and their respective coordinators was compiled through existing literature and online research. Contact information was entered into a Qualtrics distribution list, and the survey was disseminated to a total of 14 institutions in June 2016. Two reminder emails were sent approximately two weeks apart. After a six-week period, during which no further responses were anticipated, the survey was closed. All data were imported into Microsoft Excel Version 16.16.27 (Redmond, WA, USA) for analysis.

## 3. Results

Thirteen of the fourteen institutions responded to the UEOE survey, yielding a response rate of 93% (Table 1). Results from the survey are outlined below, organized into sections based on the order in which they were addressed in the survey.

### 3.1. Event Information

Survey participants were asked to identify the organizational host of their respective entomology-themed outreach event, whether it be a department within a university, a local museum, or a Cooperative Extension partner. The survey allowed for the identification of multiple hosts. The majority of respondents (85%) indicated their university department as the primary host of the event. Three respondents identified a college within a university as the host, and another three indicated a museum as the host. Other identified hosts included a university or college (2), Agriculture and Natural Resources Cooperative Extension (1), and 4-H Cooperative Extension (1).

All respondents reported that their entomology-themed outreach events occurred annually, except for one event (the University of Arkansas Insect Festival) occurring every other year. The majority (85%) were single-day events, averaging five contact hours with attendees. However, events like the Purdue University Bug Bowl and Drexel University Bug Fest spanned two days and averaged over eight contact hours per day. Most respondents (85%) indicated their events occurred during the weekend on Saturday or Sunday.

At the time of the survey, the mean (±SE) duration for which events had been established was 16 (±3) years (Table 2). The mean (±SE) attendance at these events was 7641 (±3560) individuals, while the mean (±SE) number of volunteers needed per event was 109 (±36) (Table 2). When respondents were asked to identify sources of funding (multiple funding sources could be identified), 54% selected corporate and private entities as sponsors. In addition, 54% indicated they had received financial support from their university or college. Merchandise sales (38%), admission fees (23%), and external grants (23%) were also listed as providing financial support. Across all 13 institutions, annual funding (including monetary and in-kind estimates) totaled over USD 290,000. Respondents were further asked to identify their top exhibits, with live arthropod displays, insects as food, educational displays, and performances emerging as consistent favorites. In addition, 77% of respondents indicated that they maintained live arthropod collections to support their event. Collections consisted primarily of insect and arachnid species, as well as some social insect colonies. The combined estimated value of these collections exceeded USD 299,000 annually.

### 3.2. Attendee Demographics and Information

Respondents were asked to provide an estimated age of attendees, and how far attendees were thought to have traveled to the event. The estimated age of attendees was predominantly distributed across three ranges: 20% fell between ages 5 to 8, 18% were aged 9 to 13, and 22% were parents or caregivers. Eighty-two percent of attendees were estimated to have traveled within a one-hour distance to the event.

### 3.3. Attendee Attitudes toward Arthropods and Insects

**Figure 1:**
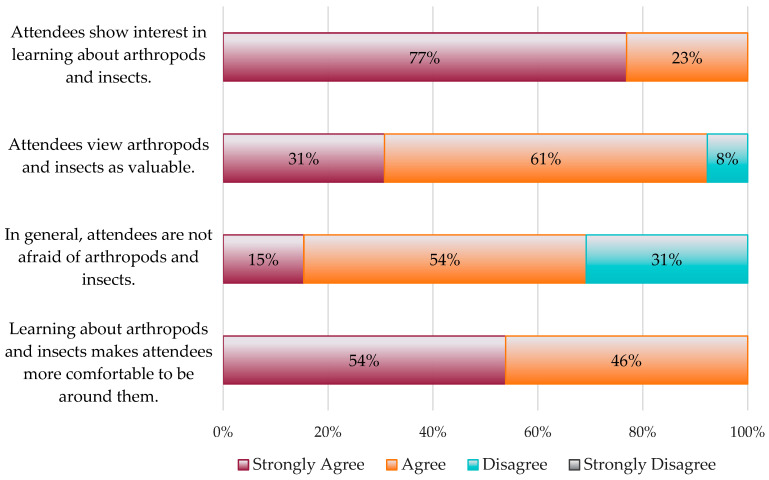
Survey responses regarding attendees’ attitudes toward entomology-themed outreach events.

A series of Likert-scale statements were used to gather information about attendees’ attitudes toward arthropods and insects. The responses to these statements were primarily positive. Overall, 77% of respondents strongly agreed that attendees showed genuine interest in learning about arthropods and insects, 92% strongly agreed or agreed that attendees viewed arthropods and insects as valuable, 69% strongly agreed or agreed that attendees were generally not afraid of arthropods and insects, and 100% strongly agreed or agreed that attendees became more comfortable around arthropods and insects as they learned about these animals (Figure 1).

### 3.4. Institutional/Community Impacts of Events

**Figure 2:**
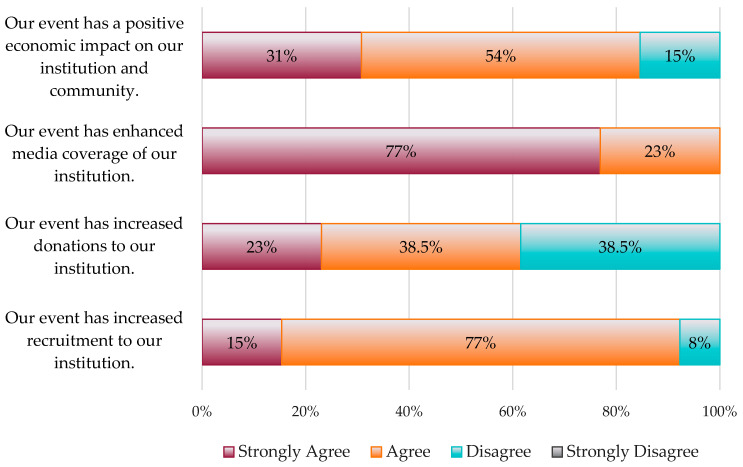
Survey responses regarding institutional impacts of entomology-themed outreach events.

A series of Likert-scale statements were similarly used to gather information about the impacts these events had on respondents’ institutions and surrounding communities. The responses to these statements generally indicated positive benefits. Overall, 77% of respondents strongly agreed their event had enhanced media coverage for their institution, 85% strongly agreed or agreed that their event had a positive economic impact on their institution and surrounding community, 61.5% strongly agreed or agreed that their event increased donations to their institution, and 92% strongly agreed or agreed their event had increased recruitment to their institution (Figure 2).

### 3.5. Interest in Collaboration

Respondents from all 13 institutions indicated that they wanted opportunities to collaborate in order to share knowledge and resources about entomology-themed outreach events. Options considered were online platforms such as Facebook, Google Groups, and website creation.

### 3.6. Open-Ended Written Responses

Respondents were also asked to provide open-ended feedback on their events. Some notable responses included:

- “People absolutely love the event. It is a community icon.” (North Carolina Museum of Natural Sciences BugFest);
- “Participants would like us to have more events throughout the year.” (University of Florida BugFest);
- “It has enhanced our department’s [public relations] in the community and within the university.” (Virginia Tech Hokie BugFest);
- “We’ve been hosting this event since 1984—we have parents who attended as children coming and bringing their own kids.” (University of Illinois Insect Fear Film Festival);
- “We have heard from students that they have been attending our event since they were children (including one of our grad students who runs the insect zoo!).” (Pennsylvania State University Great Insect Fair).

The majority of open-ended feedback included positive aspects of hosting entomology-themed outreach events.

## 4. Discussion

Our results revealed several commonalities among the entomology-themed outreach events in this study. Respondents expressed similar views regarding attendee demographics and attitudes toward arthropods and insects, community/institutional impacts, and interest in collaboration. In addition, these events shared several characteristics: they were predominately held on an annual basis, typically spanned a single day, were primarily organized by entomology departments within their respective institutions, and prominently showcased various live insect and arthropod specimens. The primary differences observed in responses pertained to records of attendance, funding sources, and the longevity of events. Event attendance varied greatly from as few as 500 attendees to as many as 40,000 attendees reported. Events drew support from diverse funding channels, including university sponsorship, corporate sponsorship, exhibit or admission fees, donations, and merchandise sales. Regarding the amount of time events had been in existence, several were relatively new, having operated for less than 6 years at the time of the survey, while many others were well established and had been operating for over 20 years.

Likert statements assessing attendee attitudes toward insects and other arthropods generally garnered positive feedback. Event coordinators indicated that attendees had mostly positive perceptions of these animals. Overall, responses indicated that attendees were interested in learning about insects and other arthropods (despite the fears some may have had), and attendees’ comfort levels increased as they learned. These observations are consistent with findings from Pitt and Shockley [13], who showed that attendees of insect zoo events had more positive views of arthropods post-event. Overall, these data suggest that entomology-themed outreach events can effectively contribute to shifting the public perception of insects. However, it is important to note that these data reflect the event coordinators’ views on attendee attitudes, which represents a limiting factor of the study. Ascertaining more accurate shifts in public perception would require further investigation.

The Likert statements assessing the impacts of these events on their institutions and surrounding communities similarly garnered positive feedback. In general, respondents indicated that their events had led to enhanced media coverage and recruitment and had increased cross-collaboration within their university and with other local and regional entities. Respondents also indicated that their event had created positive economic impacts for their institution and surrounding communities. These results are consistent with those from Hvenegaard et al. [1], who similarly showed that insect festivals can increase interactions among scientists, professionals, and the general public, and create a strong sense of community as well as positive economic impacts within localities.

Respondents unanimously indicated a clear interest and need for a collective that would allow event coordinators to connect, communicate, and share their knowledge and resources in implementing and executing entomology-themed outreach activities. This interest, along with the positive, open-ended written responses, illustrated the shared enthusiasm and passion that event coordinators had for their entomology-themed outreach events. In 2018, the Insect Festival Working Group (IFWG) was formed to facilitate networking among event coordinators [2]. The IFWG allows event coordinators to identify best practices and provides access to evaluation tools to gauge the impacts of entomology-themed outreach events [2].

Since the 2016 survey, and upon a recent review of current online information resources, there appears to have been relatively few major changes to entomology-themed outreach events, with most remaining active. While there are new exhibits at many of the events each year, significant changes in the structures, volunteers, sponsors, and attendance appear to occur less often. However, an exception arose starting in 2020, when the COVID-19 pandemic forced institutions to cancel or shift their events to alternative formats. During that time, online searches and participation in IFWG meetings confirmed that many institutions were unable to pivot to an alternative format due to the extensive planning these events require throughout the year. A few events like the North Carolina Museum of Natural Sciences BugFest, the Drexel University Bug Fest, and the Virginia Tech Hokie BugFest were able to transition their in-person events to online experiences. Planners at these institutions faced the difficult task of virtually delivering events that had traditionally occurred in person. Virginia Tech’s approach to this challenge was to build a Hokie BugFest website to house virtual exhibitor pages that functioned like online festival “booths.” Exhibitors were invited to submit pre-recorded videos, photos, downloadable at-home activities, and/or live-streamed online content for their booths. Furthermore, to honor the 10th anniversary of Hokie BugFest, the online event spanned 10 days with new content released each day.

Although the COVID-19 pandemic has subsided, online options will likely remain, presenting opportunities to broaden the geographic scope of events and offer content over an extended period of time. Offering online content can allow event coordinators to expand their audience base beyond their local communities, and further engage attendees beyond a typical single-day event. Moreover, the availability of online content can serve as a valuable supplemental resource for attendees of in-person events, allowing them to revisit and deepen their engagement long after the event concludes. Embracing online options alongside traditional in-person gatherings may enhance the overall experience and effectiveness of entomology-themed outreach events, providing a flexible and dynamic platform for knowledge sharing.

While the full effects of the COVID-19 pandemic on the future of entomology-themed outreach events still remain uncertain [16], a current examination of online information resources has revealed some adverse effects on attendance records and hosting of such events. For example, post-COVID attendance records at events like the Virginia Tech Hokie BugFest and the Arizona Insect Festival were strong but still shy of pre-pandemic levels. Other events like the Penn State University Great Insect Fair and the University of Illinois Insect Fear Film Festival postponed returning to an in-person format until 2023. Despite these disruptions in programming, positive developments have emerged. In Virginia, additional entomology-themed outreach events have become established across the state. The Virginia Tech Hokie BugFest served as a catalyst for the inception of three additional events: the Henrico County Bug Bizarre (established in 2019 and located near Richmond, VA, USA), the Southeast Virginia Hokie BugFest (established in 2021 and located in Norfolk, VA, USA), and the Big BUG Event (established in 2023 and located in Dinwiddie, VA, USA). These new events underscore the growing popularity and enthusiasm for entomology-themed engagements in the region. Moving forward, it is crucial for organizers to continue monitoring and adjusting their approaches to ensure the success and accessibility of entomology-themed outreach events, in-person and/or online. By continuing to foster collaboration and creativity, the field can continue to inspire curiosity and appreciation for insects among their audiences.

## 5. Conclusions

As existing entomology-themed outreach events continue and new events emerge, evaluating these events becomes imperative. Our results show positive impacts on attendees, their communities, and their institutions; however, the true impact of entomology-themed outreach events has yet to be realized. Further research is necessary to explore the long-term effects, reach, and effectiveness of these events in fostering public engagement with entomology and promoting scientific literacy. Such studies will provide valuable insights for organizers and educators seeking to maximize the impact and relevance of these events in the future.

## Figures and Tables

**Table 1:** Entomology-themed outreach events that participated in the UEOE survey.

Event	Website (Accessed on 27 February 2024)
University of Arkansas Insect Festival; Fayetteville, AK, USA	https://enpl.uark.edu/research/insect-festival.php
Arizona Insect Festival; Tucson, AZ, USA	https://www.arizonainsectfestival.com/
University of Florida BugFest; Gainesville, FL, USA	https://www.facebook.com/EntomologyClubUF
Georgia State Botanical Garden Insectival!; Athens, GA, USA	https://calendar.uga.edu/event/32nd_annual_insect-ival
University of Georgia Insect Zoo Open House; Athens, GA, USA	https://www.facebook.com/uga.bugdawgs
Columbus State University Insectival; Columbus, GA, USA	https://oxbow.columbusstate.edu/insectival/
University of Illinois Insect Fear Film Festival; Urbana, IL, USA	https://publish.illinois.edu/uiuc-egsa/ifff/
Indiana University Bug Fest; Bloomington, IN, USA	https://hilltop.indiana.edu/events-programs-classes/bugfest/index.html
Purdue University Bug Bowl; West Lafayette, IN, USA	https://ag.purdue.edu/department/entm/extension/bugbowl/index.html
North Carolina Museum of Natural Sciences BugFest; Raleigh, NC, USA	https://naturalsciences.org/calendar/bugfest/
Drexel University Bug Fest; Philadelphia, PA, USA	https://ansp.org/programs-and-events/festivals/bug-fest/
Penn State University Great Insect Fair; State College, PA, USA	https://ento.psu.edu/outreach/youth/great-insect-fair
Virginia Tech Hokie BugFest; Blacksburg, VA, USA	https://www.ento.vt.edu/4-H_Entomology/hokiebugfest.html

**Table 2:** Event information as reported by respondents in 2016.

Event	Year Established	Estimated Attendance	Number of Volunteers
University of Arkansas Insect Festival	1993	3000	101
Arizona Insect Festival	2011	6000	150
University of Florida BugFest	2015	500	35
Georgia State Botanical Garden Insectival!	1993	1100	50
University of Georgia Insect Zoo Open House	1986	1000	46
Columbus State University Insectival	2001	1000	30
University of Illinois Insect Fear Film Festival	1984	850	30
Indiana University Bug Fest	2013	950	18
Purdue University Bug Bowl	1990	40,000	100
North Carolina Museum of Natural Sciences BugFest	1997	31,898	500
Drexel University Bug Fest	2008	2023	12
Penn State University Great Insect Fair	1994	4000	200
Virginia Tech Hokie BugFest	2011	7020	150

## Data Availability

The data presented in this study are available upon request from the corresponding author.

## Supplementary Materials

The following supporting information can be downloaded at https://www.mdpi.com/article/10.3390/insects15050337/s1, PDF File S1: University Entomology Outreach Events Survey.
