# Supplementary material for: Comparing Entomology-Themed Outreach Events: Annual Festivals and Open Houses in the United States
Source: Insects. 2024 May 7;15(5):337. doi: 10.3390/insects15050337 (PMC11122388; doi:10.3390/insects15050337)
Supplement: Supplementary file 1 [file insects-15-00337-s001.zip › insects-2963364-supplementary.pdf]

University Entomology Outreach Events Survey

VirginiaTech
Invent the Future®

University, Entomology Outreach Events Survey

My name is Stephanie Blevins. I am a master's candidate in the Virginia Tech Department of Entomology in Blacksburg.

Our department hosts an annual event called Hokie BugFest. It is an outreach (STEM/STEAM) event that promotes the science of entomology by showcasing our research and educational programs. This will be our sixth year hosting the event.

Through literature review and web searches, I have learned your institution hosts a similar event. As part of my research study I am seeking collaborators and collecting information about these events. I have prepared a 10-15 minute survey to collect information.

The planned outcome of this work will be to share the findings through publication. I also hope to encourage further collaboration among those who participated in the survey by organizing a formal consortium.

If there is another person at your institution who is better suited to participate, please let me know so I may contact them. If you have difficulty completing the survey, please feel free to contact me with questions.

Thank you very much for your time.

Stephanie Blevins

VT IRB Number 16-587, Exempt

The purpose of this study is to collect information about university entomology outreach events. Please know that your participation is voluntary and that you may stop at any time. The data will be kept confidential, and the results may be published.

0% 100%

>>

Q1) Please indicate which event is being surveyed:

- o Arizona Insect Festiva, University of Arizona
- o BugFest, North Carolina Museum of Natural Sciences
- o Bug Fest, Drexel University
- o Bug Fest, Indiana University
- o BugFest, University of Florida
- o Bug Bowl, Purdue University
- o Hokie BugFest, Virginia Tech
- o Insectapalooza, Cornell University
- o Insect Fear Film Festival, University of Illinois
- o Insect Festival, University of Arkansas
- o Insectival, Oxbow Meadows Environmental Learning Center at Columbus State University
- o Insect-ival! Family Festival, University of Georgia State Botanical Garden
- o Insect Zoo Open House at the University of Georgia
- o The Great Insect Fair, The Penn State University
- o Other

Q2) Is the event hosted by a:
(Check all that apply.)

- o University or College (as a whole)
- o College within a University
- o Department within a University or College
- o Cooperative Extension – Ag & Natural Resources
- o Cooperative Extension – 4-H
- o Local Museum
- o Other Entity

Q3) How often do you hold the event?

- o Twice a Year
- o Annually

- Every Other Year
- Every Three Years
- Every Four Years
- Every Five Years

Q4) Which days of the week did you hold your most recent event?
(Check all that apply.)

- Monday
- Tuesday
- Wednesday
- Thursday
- Friday
- Saturday
- Sunday

Q5) How many total hours did you run your most recent event?

	0	5	10	15	20	25	30	35	40	45	50
Hours											

Q6) Including this year, how many years have you been hosting the event?

	0	5	10	15	20	25	30	35	40	45	50
Years											

Q7) Approximately how many people did it take to operate the most recent event?

	0	50	100	150	200	250	300	350	400	450	500
Volunteers											
Paid Event Staff											

Q8) What types of funding (monetary and in-kind) support the event?
(Check all that apply.)

- o Admission Fees (mandatory)
- o Exhibit Fees
- o Voluntary Donations (from attendees)
- o Merchandise Sales
- o Corporate & Private Sponsors/Donors
- o University/College Funding
- o Grants (Private & Public)
- o Other Funding

Q9) What is the cost of admission to the public?
(If admission is free, you still must move the slider forward and back to zero to proceed.)

Dollars

	0	3	6	9	12	15	18	21	24	27	30
Admission Fee											

Q10) What is the approximate value of your annual support (monetary vs. in-kind)?

Dollars

	0	10000	20000	30000	40000	50000	60000	70000	80000
Monetary									
In-Kind									

Q11) Please list five exhibits/activities that make your event attractive to the public:

1. ________ Most Significant
2. ________
3. ________
4. ________
5. ________ Least Significant

Q12) Do you keep a live arthropod collection year round to support outreach and educational efforts?

- o Yes
- o No

Q12a) Approximately how large is your live collection?

	0 20 40 60 80 100 120 140 160 180 200
Insects (# of species)	
Arachnids (# of species)	
Social Insects (# of colonies)	
Other Arthropods – myriapods, isopods, crustaceans, etc. (# of species)	

Q12b) What is the approximate value of your live arthropod collection (including their enclosures)?

	Dollars<br>0 10000 20000 30000 40000 50000
Value	

Q12c) What is the approximate cost to maintain your live arthropod collection annually?

	Dollars<br>0 20000 40000 60000 80000 100000
Personnel	
Materials & Supplies	

Q13) On a scale of 0 to 100, estimate the percentage of attendees that fall into these categories:
(Percentages indicated in each category should equal 100 when added.)

Age 0 – 4: ☐

Age 5 – 8: ☐

Age 9 – 13: ☐

Age 14 – 17: ☐

College Students: ☐

Parents: ☐

Grandparents & Senior Citizens: ☐

Total: ☐

Q14) On a scale of 0 to 100, estimate the percentage of attendees as male or female:
(Percentages indicated in each category should equal 100 when added.)

Male: ☐

Female: ☐

Total: ☐

Q15) On a scale of 0 to 100, estimate the time/effort attendees take to travel to your event:
(Percentages indicated in each category should equal 100 when added.)

Within 1 Hour Travel Distance: ☐

Within 3 Hours Travel Distance: ☐

Within 6 Hours Travel Distance: ☐

More than 6 Hours Travel Distance: ☐

Total: ☐

Q16) Please give your best estimate of how many people attended your most recent event:

____

Q17) Please indicate your opinion of the following statements.

	Strongly Agree	Agree	Disagree	Strongly Disagree
Our event provides an educational experience to the attendees.	○	○	○	○
Our event has a positive economic impact on our institution.	○	○	○	○
Our event has a positive economic impact on the community.	○	○	○	○
Our event has enhanced media coverage of our institution.	○	○	○	○
Our event has increased donations to our institution.	○	○	○	○
Our event has increased recruitment to our institution.	○	○	○	○
Our event has a positive impact on attendance at other outreach events.	○	○	○	○
Our event has increased collaborative opportunities within our institution and with other entities.	○	○	○	○

Q17a) Please share any positive or negative aspects of your event that has been conveyed to you by your participants:

____

____

____

____

____

Q18) Please indicate your opinion of the following statements.

	Strongly Agree	Agree	Disagree	Strongly Disagree
Attendees show interest in learning about arthropods and insects.	○	○	○	○
Attendees enjoy learning about arthropods and insects.	○	○	○	○
Attendees view arthropods and insects as valuable.	○	○	○	○
In general, attendees are not afraid of arthropods and insects.	○	○	○	○
Attendees tend to like some arthropods and insects more than others.	○	○	○	○
Attendees tend to like arthropods and insects more after attending our event.	○	○	○	○
Learning about arthropods and insects makes attendees more comfortable to be around them.	○	○	○	○
Attendees are interested in continuing to learn about arthropods and insects.	○	○	○	○

Q19) Would you be interested in participating in a consortium that shares knowledge and resources for entomology outreach activities? (i.e. festivals, camps, youth education, STEM/STEAM activities, bug zoos, etc.)

- o Yes
- o No

Q19a) Please provide your contact information:

Name: ____________________________________

Address: __________________________________

Address 2: ________________________________

City: _____________________________________

State: ____________________________________

Postal Code: ______________________________

Country: _________________________________

Phone #: _________________________________

E-mail: ___________________________________
